# Depression and Cognitive Dysfunction in Patients with Chronic Hepatitis C: Correlation with Viral Replication in the Peripheral Blood Mononuclear Cells and Cytokines in Serum

**DOI:** 10.3390/ijms242015351

**Published:** 2023-10-19

**Authors:** Marek Radkowski, Tomasz Kryczka, Bogna Szymańska-Kotwica, Hanna Berak, Andrzej Horban, Tomasz Pawłowski, Karol Perlejewski, Tomasz Laskus

**Affiliations:** 1 Department of Immunopathology of Infectious and Parasitic Diseases, Medical University of Warsaw, 02-106 Warsaw, Poland; mradkowski@wum.edu.pl (M.R.); kperlejewski@wum.edu.pl (K.P.); 2 Department of Development of Nursing and Social and Medical Sciences, Medical University of Warsaw, 01-445 Warsaw, Poland; tomasz.kryczka@wum.edu.pl; 3 Outpatient Clinic, Warsaw Hospital for Infectious Diseases, 01-201 Warsaw, Poland; bogna.szymanska@gmail.com (B.S.-K.); hanna.berak@zakazny.pl (H.B.); 4 Department of Adult Infectious Diseases, Medical University of Warsaw, 01-201 Warsaw, Poland; 5 Department of Psychiatry, Wroclaw Medical University, 50-367 Wroclaw, Poland; tomasz.pawlowski@am.wroc.pl

**Keywords:** HCV, depression, cognitive, chronic, hepatitis

## Abstract

Chronic hepatitis C virus (HCV) infection is commonly associated with depression and cognitive dysfunction, the cause of which could be related to the HCV neuroinvasion and/or state of chronic inflammation. Viral sequences and proteins were previously detected in the brain and since blood leukocytes can cross the blood–brain barrier, they could provide viral access to the CNS. Eighty chronic hepatitis C patients were tested for viral replication in PBMCs (detection of the HCV RNA-negative strand) and serum cytokines. Depression was assessed by the Beck Depression Inventory (BDI), neuroticism by the Eysenck Personality Inventory (N/EPO-R), and anxiety by the State-Trait Anxiety Inventory (STAI) while neurocognitive testing included the Wisconsin Card Sorting Test (WCST), Ruff Figural Fluency Test (RFFT), California Verbal Learning Test (CVLT), and Grooved Pegboard Test (GPT). The HCV RNA-negative strand was detected in PBMCs from 24 (30%) patients and these patients had significantly higher BDI scores (median 12.5 [IQR] 6.3–20.5 vs. median 8.00 [IQR] 3–12; *p* = 0.013). Both depression and anxiety correlated positively with IL-8 while cognitive flexibility, executive function, problem-solving skills, memory, and motor functioning correlated negatively with some proinflammatory cytokines. Our findings suggest that due to chronic HCV infection, the brain function is negatively affected by both viral replication in PBMCs and by the immune activation state.

## 1. Introduction

Chronic hepatitis C virus (HCV) infection is commonly associated with depression and cognitive dysfunction [1,2,3]. The cause of these problems was largely attributed to liver dysfunction and the awareness of chronic illness until Forton and colleagues provided evidence for the likely biological basis of HCV-related brain effects. In proton magnetic-resonance spectroscopy (^1^H MRS), patients with hepatitis C demonstrated elevations in choline/creatine ratios in basal ganglia and white matter, which were not present in patients with hepatitis of another etiology or healthy controls [2,4]; notably, in hepatic encephalopathy, the choline ratios were depressed [5]. Similar findings were since reported by other groups, strengthening the argument that depression and neurocognitive disturbances are directly related to HCV infection and not secondary to liver disease [6,7]. Importantly, many of the same changes are encountered in HIV-infected patients [8,9] and HIV is known to directly infect and injure the central nervous system (CNS), leading to a wide spectrum of neuropsychiatric symptoms (so-called neuroAIDS), including a plethora of neurocognitive disorders, such as dementia, minor neurocognitive disorder, depression, and anxiety [10].

The results of these studies, and some similarities to the effects of HIV infection, led to speculation that HCV could be neuroinvasive and neurotropic; this suspicion was confirmed when negative-strand HCV RNA, which is a viral replicative intermediate, was detected in an autopsy of brain tissue [11] and two subsequent studies demonstrated the presence of HCV proteins in brain tissue by Western Blotting and/or immunostaining [12,13]. Similarly to findings in HIV-infected patients, brain-derived HCV variants could represent a separate compartment as they are often distinct from those circulating in the blood [11,14]. Furthermore, some studies demonstrated a close relationship between the HCV sequences present in the CNS and those found in peripheral blood mononuclear cells (PBMCs) and lymph nodes [11,14,15], which suggests that the virus could gain access via infected leucocytes in a process similar to the one postulated for HIV infection (the so-called ‘Trojan horse’ phenomenon) [16]. HCV is considered to be lymphotropic—several groups of researchers have detected viral replicative forms in PBMCs, lymph nodes, and bone marrow and it was also demonstrated that viral genomic sequences present in extrahepatic sites often differ from those found in the serum and liver [17,18,19]. In chimpanzees, the same minor variants of the HCV strain H77, which were selected in lymphoblastoid cells in vitro, were found to be replicating in vivo in PBMCs when the animals were inoculated with the same parental strain [20].

The aim of this study was to determine whether depression and neurocognitive deficiencies in chronic HCV infection correlate with the presence of replication in PBMCs. Furthermore, since there is mounting evidence that chronic inflammation in the brain and body may contribute to the development and maintenance of depressive symptoms [21], we tested the serum levels of a number of cytokines. Viral extrahepatic replication was determined by the detection of viral negative strand and cytokines were measured by a commercially available assay.

## 2. Results

The HCV RNA-negative strand was detected in PBMCs from 24 (30%) out of 80 analyzed patients. Patients in whom the viral negative strand was detected did not differ from those in whom it was not detected with respect to viral load (median 1.07 × 10^6^, interquartile range [IQR] 2.56 × 10^5^–2.7 × 10^6^ vs. median 9.91 × 10^5^ [IQR] 1.58 × 10^5^–2.9 × 10^6^) but were less likely to have advanced liver disease as the METAVIR score F3/F4 was found in 35% of patients in the former and 53% of patients in the latter group. However, this difference did not reach statistical significance (*p* = 0.26). Interestingly, patients with evidence of HCV replication in their PBMCs had significantly higher IL-8 serum levels (median 26.3 [IQR] 19.0–50.1 vs. median 22.5 [IQR] 14.8–31.9; *p* = 0.025).

**Figure 1:**
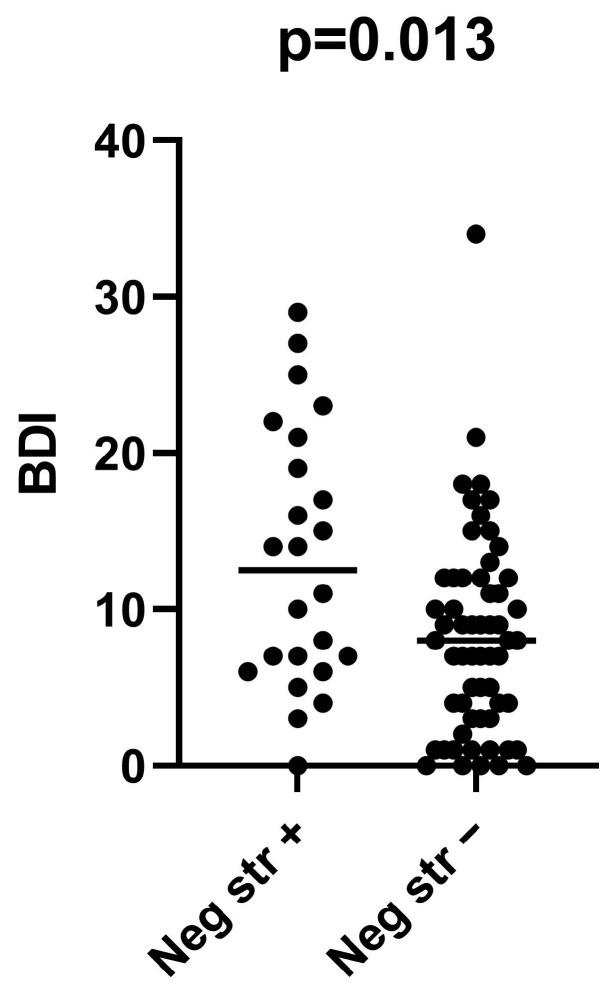
Depression in patients with the chronic HCV infection in relation to the presence or absence of the HCV RNA-negative strand in the PBMCs. Depression was measured by the Beck Depression Index (BDI) and the HCV RNA-negative strand (viral replicative form) was detected by a strand-specific assay in 24 out of 80 patients. Horizontal lines represent median values. Data were compared using the nonparametric Mann–Whitney U test.

The two groups of patients did not differ with respect to the results of any of the cognitive tests, nor with respect to the level of neuroticism or anxiety. However, there was a significant difference regarding the depression scores (median 12.5 [IQR] 6.3–20.5 vs. median 8.00 [IQR] 3–12; *p* = 0.013) in these two groups (Figure 1).

**Figure 2:**
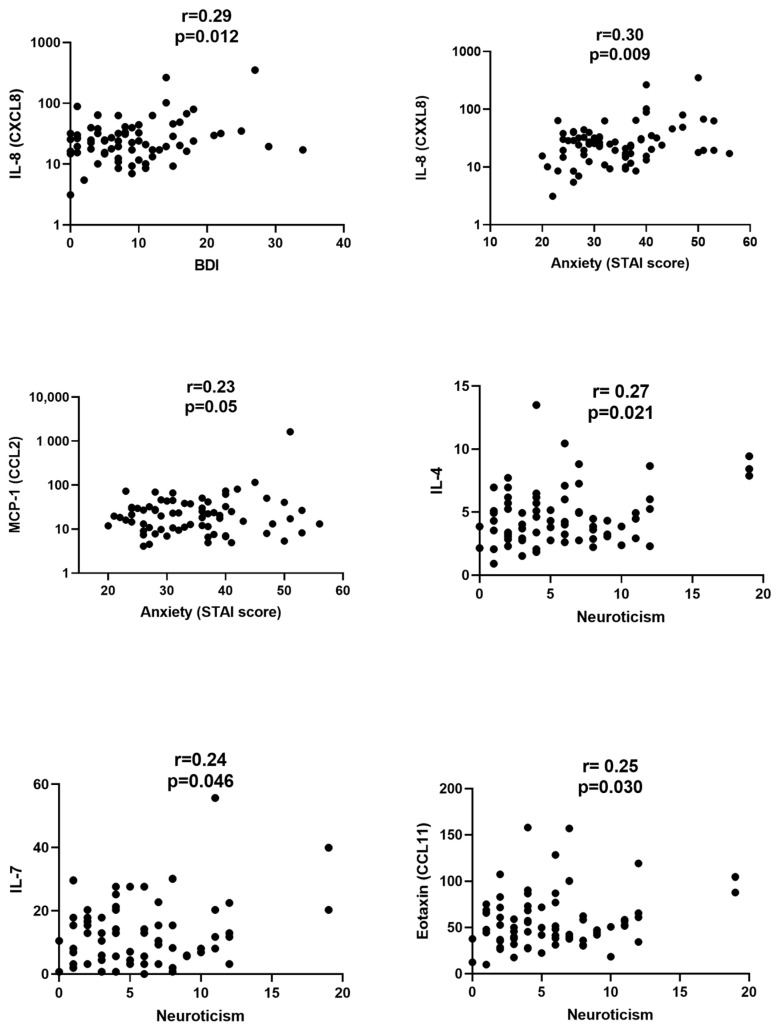
Correlation between the cytokine/chemokine levels in the serum and depression, anxiety, and neuroticism. Neuroticism was assessed by the Revised Eysenck Personality Test while depression and anxiety were measured by the Beck Depression Index (BDI) and State-Trait Anxiety Inventory (STAI), respectively.Detailed comparisons are shown in Table 1 and only correlations that were statistically significant are shown in the figure. Correlation coefficients r and statistical significance are provided in the panels. Cytokines are provided as pg/mL.

Correlations between the scores for depression, anxiety, and neuroticism and the levels of cytokines in serum are shown in Table 1 and Figure 2. The BDI scores showed a moderate correlation with IL-8 (r = 0.29; *p* = 0.012) and borderline with GM-CSF (r = 0.21; *p* = 0.08); meanwhile, anxiety correlated with IL-8 (r = 0.30; *p* = 0.009) and borderline with IFN-gamma (r = 0.21; *p* = 0.06) and MCP-1 (r = 0.23; *p* = 0.051). When neuroticism levels were correlated with the cytokines’ concentrations, statistically significant relationships were found for IL-4 (r = 0.27; *p* = 0.02), IL-7 (r = 0.24; *p* = 0.046), and Eotaxin (r = 0.25; *p* = 0.03).

**Figure 3:**
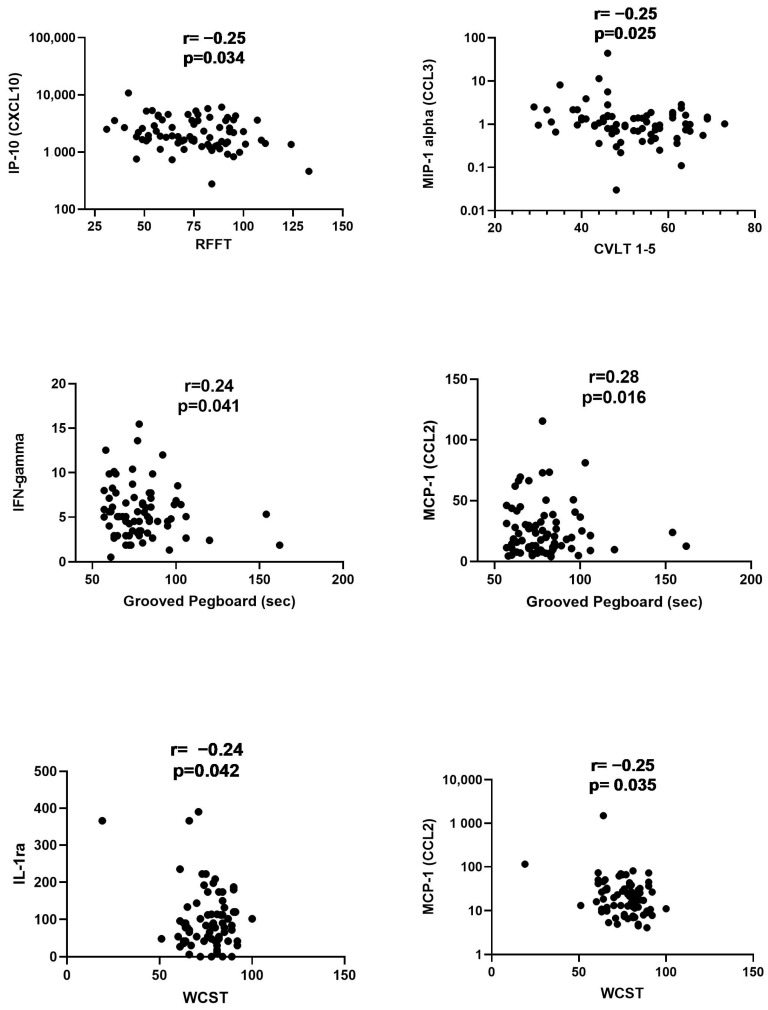
Correlation between the cytokine/chemokine levels in serum and the results of Ruff Figural Fluency Test (RFFT), California Verbal Learning Test (CVLT), Grooved Pegboard and Wisconsin Card Sorting Tests (WCST). The RFFT is shown as a number of unique designs and CVLT represents sum of immediate recall from Trial-1 to Trial-5. The results of The Grooved Pegboard Test are shown in seconds while the WCST is represented as the total number of correct responses. Detailed comparisons are shown in Table 2 and only correlations that were statistically significant are shown in the figure. Correlation coefficients r and statistical significance are provided in the panels. Cytokines are provided as pg/mL.

Correlations between the cytokine levels and neurocognitive scores are presented in Table 2 and Figure 3. The RFFT, which was analyzed only with respect to the number of unique designs, showed a weak negative correlation with IP-10 (r = −0.25; *p* = 0.034) only; meanwhile, CVLT scores representing the sum of immediate recall from Trial-1 to Trial-5 correlated with MIP-1alpha (r = −0.25; *p* = 0.025). Motor functioning, as assessed by the Grooved Pegboard Test (GPT) time, correlated with IFN-gamma (r = 0.24; *p* = 0.41) and MCP-1 (r = 0.28; *p* = 0.016). When the results of the WCST were correlated with cytokine levels, the numbers of correct responses showed a moderate negative correlation with IL-1ra (r = −0.24; *p* = 0.042) and MCP-1 (r = −0.25; *p* = 0.035) while other responses (errors, perseverative responses, nonperseverative errors) did not show any statistically significant correlations 

To further analyze factors affecting depression, multiple linear regression was performed, taking into account the presence of the viral negative strand in PBMCs, IL-8, and GM-CSF. The presence of the viral negative strand in PBMCs and the level of IL-8 showed statistically significant relationships with the BDI scores (regression coefficient 3.89 [95% CI] 0.53–7.25; *p* = 0.024) and 0.037 [95% CI] 0.007 to 0.066; *p* = 0.016, respectively). Multicollinearity was small (R^2^ with other variables was 0.10 for the presence of a negative strand and 0.07 for IL-8 concentration).

## 3. Discussion

Ours is the first study suggesting the potential role of extrahepatic replication in HCV-associated depression. There is strong evidence that the HCV can be neurotropic, as demonstrated in postmortem studies finding viral replicative forms and viral proteins in brain tissue [11,12,13]. Furthermore, brain-derived HCV variants are often distinct from those circulating in the blood and may even contain tissue-specific adaptations in the internal ribosomal entry site (IRES) [11,14]. The cells harboring the HCV were identified by two independent groups as astrocytes and macrophages/microglia cells [12,13] and it was subsequently shown that the infected cells were activated [22]. In the latter study, which analyzed CD68+ cells isolated from autopsy brain tissue, it was found that mRNA transcripts for key immune activation cytokines were higher in HCV-positive patients than in HCV-negative control patients and even in HCV-positive compared to HCV-negative CD68 cells in the same patient. Within the population of PBMCs, the cells harboring the replicating virus have been identified mainly as monocytes/macrophages and B cells; although, T-cells can be infected as well, particularly in terms of long-lasting infections [23,24]. Since some monocyte family members are constantly being replaced as part of normal physiology [25,26] and all basic groups of leukocytes have the ability to enter the brain under certain conditions [27], it is highly probable that infected PBMCs constitute the vehicle for the transmission of the HCV from the periphery to the brain; thus, the presence of the viral negative strand in these cells could constitute a surrogate marker of viral replication in the CNS.

Our findings that the BDI score was higher in the presence of extrahepatic viral replication in PBMCs and correlated with the IL-8 serum levels are compatible with the inflammatory theory of depression. There is mounting evidence that any chronic inflammatory process could affect the neurotransmission, neuroplasticity, and neurogenesis of mood-regulating brain regions [21,28]. The proinflammatory cytokines can increase cortisol synthesis by activating the hypothalamic–pituitary–adrenal axis and can also activate the tryptophan–kynurenine pathway, leading to the synthesis of the neurotoxic N-methyl-D-aspartate (NMDA) glutamate agonist quinolinic acid and 3-hydroxykynurenine [28]. The distribution of the tryptophan–kynurenine pathway in the brain is concentrated mainly in the astrocytes and microglia [29]. Increased inflammatory cytokines in microglial cells can also influence the neuronal reuptake of monoamine neurotransmitters by regulating neuronal mitogen-activated protein kinase (MAPK), leading to an increased surface expression of monoamine transporters on neurons [30]. Furthermore, there is experimental evidence that the widely used and effective selective serotonin reuptake inhibitor (SSRI) antidepressants have yet another biological effect as they reduce the release of cytokines from activated macrophages and microglia [31] and non-steroidal anti-inflammatory drugs, such as the cyclooxygenase-2 inhibitor celecoxib, can enhance the response to standard antidepressant treatment [32]. Similarly, a tetracycline antibiotic, minocycline, an inhibitor of microglial inflammatory activation, has shown promising antidepressant activity in the treatment-resistant depression patients [33].

Increased proinflammatory cytokines in circulation have the propensity to repress the expression of several tight-junction proteins of the blood–brain barrier (BBB), thus promoting the passage of circulating proinflammatory cytokines and depression-like behaviors in mice [34]. However, although increased levels of proinflammatory cytokines in the plasma and CSF of depressed patients have been reported, the discriminative power of cytokine concentrations between depressed and non-depressed people is weak and they have also been shown to be altered in other psychiatric disorders [35,36]. Confusingly, the same cytokine can be produced by multiple cell types and the same cell can produce various cytokines. Importantly, many patients suffering from Major Depressive Disorder (MDD) have no evidence of increased inflammatory cytokine activity and most people with evidence of increased inflammation do not suffer from depression [37,38]. While the exogenous administration of cytokines such as interferon-alpha can trigger depression, the majority of treated patients remain unaffected [39]. These observations led to speculation that there may be a specific subtype of depression (so-called inflammatory cytokine-associated depression) that is uniquely associated with inflammatory cytokines and which may even require different treatment [37].

There is ample literature demonstrating evidence of neurocognitive defects in patients with the chronic HCV infection, usually in the form of the mild impairment of attention, concentration, and working memory [40]. However, it is difficult to determine whether these impairments are attributed to the virus itself or are secondary to liver injury, an inflammatory state, awareness of chronic disease, or possibly to a mixture of several factors. A particular problem is posed by minimal hepatic encephalopathy (MHE), which can affect the results of neurocognitive testing. Hilsabeck and colleagues reported that cognitive test performance was associated with the fibrosis stage on liver biopsy and, thus, was likely influenced by MHE; however, impairments in attention and concentration were evident, even in patients with minor hepatic injury [41]. Weissenborn et al. [6] reported that in comparison to the healthy controls, the patients with the HCV infection showed evidence of cognitive impairment, primarily attention, higher executive functions, higher levels of anxiety, and depression; these results were associated with MRS changes, thus eliminating the possibility of the confounding effect of MHE. In another study limited to patients with histologically mild liver disease, selective impairments of concentration and working memory were reported that were not seen to the same degree in patients who had recovered from HCV infection [2]. In yet another study conducted on a very homogenous cohort of iatrogenically infected women, PCR-positive women had impaired memory and attention compared to normal controls and the PCR-negative group [42]. However, some other studies have found no association between HCV infection and impaired cognitive function [3,43].

In contrast to these previous studies, our analysis did not address the question of whether HCV-infected patients have neurocognitive deficits, nor were our patients compared to controls; however, we correlated the neurocognitive test results with serum cytokine levels and the presence of extrahepatic HCV replication in PBMCs. Our findings suggest that the inflammatory process, as reflected by the cytokine levels, has some impact on neurocognitive function in chronic hepatitis C. However, while clinically overt encephalopathy was excluded, MHE was likely to be a factor since a large proportion of our patients had advanced liver fibrosis. The association between cytokines and neurocognitive dysfunction has been most extensively studied in the setting of mild cognitive impairment (MCI), which is a common precursor of Alzheimer’s disease (AD). In their comprehensive overview of 118 research articles on cytokine and other inflammation-associated protein levels in the plasma, serum, and cerebrospinal fluid (CSF) of patients with AD and MCI, Brosseron et al. [44] concluded that while increased levels of several cytokines are possible indicators of neuroinflammation, many reports are controversial or inconclusive. However, several studies reported on the increased levels of IFN-gamma, IP-10, MCP-1, and MIP-1alpha and these very cytokines correlated negatively with neurocognitive function among our patients.

In a previous study [45], we found a correlation between the PBMC activation state, as reflected by the levels of mRNA transcripts of several cytokines, and depression and neuroticism in patients after treatment with pegylated interferon and ribavirin. However, this study was limited to 24 patients and neither the viral negative-strand nor neurocognitive function were analyzed. Furthermore, the administration of interferon could have affected the results as it could have activated inflammatory responses in the brain [46].

The effect of HCV infection on the brain is still hypothetical but could share some similarities with HIV, which infects the same types of cells: brain macrophages and microglia. These cells, in turn, secrete proinflammatory cytokines, which are believed to play an important role in the pathogenesis of neuroAIDS [47].

The finding of higher IL-8 serum levels in patients with HCV replication in the PBMCs was not unexpected as we have previously reported that the infection of primary human macrophages with the hepatitis C virus in vitro results in the induction of IL-8 [48]. It was shown that the core protein strongly upregulates IL-8 transcription through the activation of NF-κB and that naturally occurring amino acid changes in the C terminus of the core protein affect this ability [49,50]. Thus, it is likely that the infected lymphoid cells were the source of elevated IL-8 in our patients.

In summary, we found a correlation between the level of depression and the presence of HCV replication in PBMCs and between neurocognitive function and cytokine serum levels. These findings suggest that in patients with the chronic HCV infection, brain function is influenced by both viral replication in PBMCs and by the overall immune activation state.

## 4. Materials and Methods

### 4.1. Patients

The current analysis included 80 patients who were being evaluated at the Outpatient Clinic of the Municipal Hospital for Infectious Diseases between January 2019 and February 2020 as candidates for antiviral treatment. Eligible patients for this study were at least 18 years old, had detectable plasma HCV RNA levels, and were required to have no history of decompensated liver disease and/or liver encephalopathy. Furthermore, patients who were active alcohol or drug abusers were excluded from this study. Enrolled patients underwent psychological testing and biological samples, consisting of sera and peripheral blood mononuclear cells (PBMCs); they were collected at the same time point. All testing preceded the initiation of antiviral therapy and separated PBMCs and serum samples were kept at −80 °C until analysis.

There were 39 women (49%) and 41 men (51%), their median age was 46.5 years (range 28–83 years) and the median duration of infection was 6.0 years (range 1–43 years). The median pretreatment HCV viral load was 9.94 × 10^5^ IU/mL (range 1.32 × 10^2^–1.09 × 10^7^). Liver fibrosis was assessed by FibroScan and a 5-point METAVIR scale grading where F0/F1 represents no or minimal fibrosis, F2 moderate fibrosis, F3 severe fibrosis, and F4 cirrhosis [51]. Among the 64 patients with whom this analysis was conducted, 23 were classified as F0/F1, 10 as F2, 10 as F3, and 21 as F4.

Informed consent was obtained from each patient included in this study and the study protocol followed the ethical guidelines of the 2013 Declaration of Helsinki and was approved by the Bioethical Committee of the Medical University of Warsaw (Approval Number KB/77/A/2015).

### 4.2. Detection of the HCV RNA-Negative Strand in PBMC

Peripheral blood mononuclear cells (PBMCs) (3 × 10^5^–10^6^ cells) were isolated from the blood by centrifugation over a density gradient and RNA was extracted by a modified guanidinium thiocyanate-phenol/chloroform technique using a commercially available kit (TRIZOL LS, Gibco/BRL, Mississauga, ON, Canada). The strand specificity of RT-PCR was assured by conducting the reverse transcription step at 65 °C with the help of the thermostable enzyme Tth, as described previously [17,52]. This assay was found to be capable of detecting approximately 100 genomic equivalent (eq) molecules of the correct strand while unspecifically detecting ≥10^7^–10^8^ genomic eq of the incorrect strand.

### 4.3. Detection of Cytokines and Chemokines in Serum

Serum samples were tested for the concentration of a panel of cytokines reflecting the immune system activation status; commercially available assay was used (Bio-Plex Pro Human Cytokine 27-plex Assay; Bio-Rad Laboratories Inc., Hercules, CA, USA).

### 4.4. Psychological Evaluation

The patients’ mental states were assessed by psychological clinical examination and psychometric tests. Depression was measured by the self-administered multiple-choice Beck Depression Inventory (BDI) [53] and neuroticism was evaluated by the Eysenck Personality Inventory (N/EPO-R), which is also a self-assessment test [54]. Anxiety was assessed by the State-Trait Anxiety Inventory (STAI), which is a commonly used measure of trait and state anxiety [55] while cognitive function was assessed by the Wisconsin Card Sorting Test (WCST) [56], Ruff Figural Fluency Test (RFFT) [57], and California Verbal Learning Test (CVLT) [58]. The WCST measures cognitive flexibility, executive function, and problem-solving skills; responses are classified in various ways: (A) correct; (B) errors; (C) perseverative responses; and (D) nonperseverative errors. The RFFT, which assesses non-verbal fluency within the domain of executive functioning, was recorded as a number of unique designs and the CVLT, which assesses verbal learning and memory, was recorded as the sum of immediate recall from Trial-1 to Trial-5 (the total score is the sum of correct responses to the five presentations). Motor functioning was assessed by the Grooved Pegboard Test (GPT) [59]; however, some research indicates that performance on this test may also reflect cognitive factors, particularly attention and executive functioning.

### 4.5. Statistical Analysis

Continuous variables were summarized as medians and categorical as frequencies and percentages. The Mann–Whitney U test was used to compare continuous variables among independent groups and correlations were calculated using Spearman’s correlation test or Pearson’s correlation test if data passed the D’Agostino-Pearson test for normality. When *p* ≤ 0.05, this was considered to be statistically significant. In addition, *p*-values corrected for the false discovery rate (FDR) were calculated using the Benjamini–Hochberg procedure (*q*-value ≤ 0.1). Calculations were performed using GraphPad Prism version 9.50 for Windows (GraphPad Software, San Diego, CA, USA).

## Figures and Tables

**Table 1:** Correlation between the Beck Depression Index (BDI) scores, anxiety levels assessed by the State-Trait Anxiety Inventory (STAI), neuroticism levels assessed by the Eysenck Personality Inventory (N/EPO-R), and the serum levels of various cytokines in 80 patients with chronic hepatitis C.

Cytokine/ Chemokine	BDI		STAI		Neuroticism	
	Correlation Coefficient	Statistical Significance ^a^	Correlation Coefficient	Statistical Significance	Correlation Coefficient	Statistical Significance
IL-1ra	−0.06	NS	0.10	NS	−0.19	NS
IL-1β	−0.08	NS	−0.02	NS	−0.14	NS
IL-2	−0.10	NS	−0.01	**NS**	−0.13	NS
IL-4	−0.05	NS	−0.04	NS	0.27	**0.02 (0.37) ^b^**
IL-7	0.02	NS	−0.05	NS	0.24	**0.046 (0.38)**
IL-8	0.29	**0.012 (0.28)**	0.30	**0.009 (0.21)**	0.18	NS
IL-9	−0.04	**NS**	0.14	NS	0.08	NS
IL-13	−0.10	NS	−0.12	NS	−0.06	NS
IL-17	−0.12	NS	−0.12	NS	0.10	NS
TNF-α	−0.13	**NS**	−0.11	NS	0.04	**NS**
IFN-γ	0.13	NS	0.21	0.06 (0.51)	−0.03	NS
IP-10/CXCL10	−0.07	NS	0.09	NS	−0.05	NS
GM-CSF	0.21	0.08	0.06	NS	0.03	NS
G-CSF	−0.04	NS	0.07	NS	0.15	NS
FGF-basic	0.16	NS	0.07	NS	−0.01	NS
PDGF-BB	−0.10	NS	−0.04	NS	−0.06	NS
MCP-1/CCL2	0.11	NS	0.23	**0.05 (0.51)**	−0.02	NS
MIP-1α/CCL3	0.029	NS	0.09	NS	0.12	NS
MIP-1β/CCL4	0.12	NS	0.06	NS	0.00	NS
RANTES/CCL5	−0.14	NS	−0.03	NS	−0.07	NS
Eotaxin	−0.04	NS	−0.10	NS	0.25	**0.03 (0.37)**

^a^ Only *p* values ≤ 0.1 are listed, *p* values ≤ 0.05 are shown in bold. ^b^ The *p* values corrected for the false discovery rate are shown in parentheses.

**Table 2:** Correlation between the results of the Ruff Figural Fluency Test (RFFT), California Verbal Learning Test (CVLT), Grooved Pegboard Test and levels of various cytokines in 80 Patients with chronic hepatitis C.

Cytokine/ Chemokine	RFFT ^a^		CVLT 1–5 ^b^		Grooved Pegboard Test	
	Correlation Coefficient	Statistical Significance ^c^	Correlation Coefficient	Statistical Significance	Correlation Coefficient	Statistical Significance
IL-1ra	−0.06	NS	−0.10	NS	−0.07	NS
IL-1β	−0.08	NS	0.16	NS	0.09	NS
IL-2	0.01	NS	−0.12	NS	0.13	NS
IL-4	0.10	NS	0.05	NS	−0.07	NS
IL-7	0.01	NS	0.08	NS	0.00	NS
IL-8	−0.18	NS	−0.08	NS	0.13	NS
IL-9	0.05	NS	−0.02	NS	−0.05	NS
IL-13	−0.19	NS	−0.17	NS	−0.03	NS
IL-17	−0.12	NS	−0.13	NS	−0.05	NS
TNF-α	0.03	NS	−0.05	NS	−0.13	**NS**
IFN-γ	0.10	NS	0.17	NS	0.24	**0.041 (0.48) ^d^**
IP-10/CXCL10	−0.25	**0.034 (0.74)**	−0.09	NS	0.10	NS
GM-CSF	0.06	NS	0.17	NS	0.02	NS
G-CSF	−0.02	NS	−0.16	NS	0.02	NS
FGF-basic	−0.15	NS	−0.05	NS	0.04	NS
PDGF-BB	0.05	NS	0.05	NS	−0.19	NS
MCP-1/CCL2	−0.16	NS	−0.04	NS	0.28	**0.016 (0.36)**
MIP-1α/CCL3	−0.14	NS	−0.25	**0.025 (0.56)**	0.00	NS
MIP-1β/CCL4	0.01	NS	0.04	NS	−0.17	NS
RANTES/CCL5	−0.07	NS	0.04	NS	−0.08	NS
Eotaxin	0.04	NS	0.02	NS	−0.03	NS

^a^ A unique design number. ^b^ Sum of immediate recall from Trial-1 to Trial-5. ^c^ Only *p* values ≤ 0.1 are listed, *p* values ≤ 0.05 are shown in bold. ^d^ The *p* values corrected for the false discovery rate are shown in parentheses.

## Data Availability

The data presented in this study are available on request from the corresponding author.
